# Abdominal Fibroma Simulating Splenomegaly

**Published:** 1908-03

**Authors:** David A. Alexander


### ABDOMINAL FIBROMA SIMULATING SPLENOMEGALY.

BY

DAVID A. ALEXANDER, M.B.

---

On August 19th I was consulted by a man, J.P., aged 61, feeble, pale and thin, who complained of indigestion and pain in the stomach, and of health impaired for upwards of six months.

On proceeding to inspect the abdomen, it was at once obvious that the left side was the seat of a tumefaction, the contours of which were those of an enlarged spleen. Smooth and firm, emerging from the left hypochondrium, and filling the flank, its mesial border lay in a line from the anterior superior iliac spine to the epigastrium, and presented an edge with a double indentation resembling splenic notching, and splenic dulness was co-extensive with it in all dimensions. Concomitant symptoms of leucocythæmia were wanting, there had been no hemorrhages, there were no sensory defects ; other organs were normal, glands were not enlarged, nor was there tenderness over bones.

The patient's previous history was without bearing on the existing symptoms, except that he was a painter, and had apparently once suffered from lead colic.

Four days later occurred an incident not unusual in the progress of splenic leucocythæmia, violent diarrhœa, ushered in by a rigor and a rise of temperature, accompanied by a hemorrhage from the bowel, and tenderness and œdema of the parietes over the swelling. With the subsidence of the attack the latter underwent no diminution, but was rather larger.

On September 1st the blood was examined, and all the changes characteristic of leucocythæmia were found to be absent. From that time until the patient's death on November 22nd the course of the complaint was that of increasing asthenia. As food was refused, and the abdominal walls grew thinner, certain bosses were to be felt upon the surface of the swelling, and its conformity to the spleen was somewhat lessened.

A partial autopsy permitted the nature of the growth to be ascertained. Underlying the abdominal walls were some coils of emptied intestines superficial and adherent to it ; others, also adherent, were pushed aside. The spleen was found unaltered in its usual site, the tumour in contact with it, as it was also with the left kidney. In removing it the abdominal parietes were exposed behind, its origin apparently being from the fibrous tissues underlying the peritoneum. No metastases were discoverable.

The tumour was firm and heavy, and showed on section several cavitations, where, as with the bosses on the surface, the tissues were breaking down. The microscope showed these tissues to be fibrous ; no sarcomatous cells were observed.

Had the patient come earlier under observation the tumour, like the desmoids of the anterior abdominal walls, might have been removed by surgical measures.

It remained, however, to present a difficulty in the diagnosis, firstly from leucocythæmia, and after that from other splenic enlargements, in which the evidence of the blood was not final.

The preparation of sections and the examination of the blood were the work of Professor Walker Hall.

